# Complex Management of Septic Joint and Cutaneous Nocardiosis in an Immunocompromised Patient: A Case Report

**DOI:** 10.7759/cureus.57810

**Published:** 2024-04-08

**Authors:** Annalee Mora, Opeyemi Oyenusi, Serfraz Abbasi, Nikolay Mitzov, Amrita Randhawa

**Affiliations:** 1 Internal Medicine, HCA Healthcare/USF Morsani College of Medicine GME/HCA Florida Oak Hill Hospital, Brooksville, USA

**Keywords:** cutaneous nocardiosis, immunocompromise, septic joint, nocardiosis, nocardia brasiliensis

## Abstract

*Nocardia*, an opportunistic, gram-positive, catalase-positive, rod-shaped bacterium found in soil and water, is known to cause infections in humans, predominantly among immunocompromised individuals, through inhalation or direct inoculation. This report details a rare case of a septic joint caused by *Nocardia brasiliensis*, which subsequently led to cutaneous involvement, in a patient with multiple underlying health issues. The management of this case was complicated by the patient’s extensive medical history, including diabetes, chronic urinary tract infections, and recent surgical procedures, which necessitated a nuanced approach to antibiotic therapy. The treatment regimen underwent several adjustments in response to concurrent infections in other organs and the emergence of multidrug-resistant organisms. Despite an expanded arsenal of therapeutic options for *Nocardia *infections, treating such infections remains challenging due to potential adverse outcomes, particularly in immunocompromised patients prone to infection relapse. This case underscores the complexities involved in diagnosing and managing *Nocardia* infections and highlights the importance of tailored antibiotic therapy in achieving favorable outcomes while minimizing the risk of relapse.

## Introduction

*Nocardia*, a genus of gram-positive, rod-shaped bacteria found in soil and water, comprises over 100 species capable of infecting humans [1]. Typically, *Nocardia* infections are acquired through inhalation or direct inoculation, predominantly affecting the lungs and skin of individuals with weakened immune systems [2]. Infections of the joints by *Nocardia* are rare, as illustrated by the case discussed here. *Nocardia asteroides *is the species most commonly linked to human diseases [1]. By contrast, *Nocardia brasiliensis* is known to cause cutaneous and localized infections following trauma or through skin lesions [3], posing a significant risk to immunocompromised patients due to increased vulnerability to relapse or disseminated infections. The treatment for septic arthritis caused by *Nocardia brasiliensis* typically involves a combination of surgical debridement and antibiotic therapy [4]. However, the effective management of this infection presents challenges. These include the variability among *Nocardia* species, their differing geographical prevalences, the bacteria’s taxonomy that influences their antimicrobial resistance patterns [5], and the patient’s ongoing immunosuppression. Addressing this case required careful consideration of the balance between administering effective antibiotics for *Nocardia* infections and the risk of exacerbating secondary diseases due to the compromised state of the patient’s healthy microbiome.

## Case presentation

A 76-year-old man with a history of type 2 diabetes, hypertension, peripheral artery disease, chronic lumbar radiculopathy, recurrent urinary tract infections (UTIs), neurogenic bladder managed with a chronic suprapubic catheter, and a recent transurethral resection of the prostate (TURP) was admitted due to generalized weakness, altered mental status, right knee pain, subjective fevers, and shortness of breath persisting for two weeks. His family reported a fall from his bed four days before admission, after which he was unable to get up for two hours until his wife discovered him. The patient experienced increased difficulty walking due to worsening lower extremity weakness. He did not report headaches, dizziness, chest pain, chills, nausea, vomiting, abdominal pain, blood in stool (hematochezia), or urine (hematuria).

Upon examination, his blood pressure was 180/80 mmHg, heart rate 97 beats per minute, respiratory rate 26 breaths per minute, body temperature 36.6°C, and oxygen saturation 95% on 2 L of oxygen per minute. He was alert and oriented, without distress. Examination of the head showed no trauma or abnormalities, with no visual field defects. His cardiovascular examination was normal without murmurs, but decreased breath sounds were noted bilaterally. The lower extremities were tender with 1+ pitting edema below the knees, but without cyanosis and lesions and with intact bilateral sensory function.

Laboratory tests revealed low hemoglobin and hematocrit levels and significant leukocytosis (24 x 103/µL). Serum electrolytes, serum urea nitrogen, creatinine, troponins, lactic acid, and creatine kinase levels were within reference ranges. Elevated C-reactive protein (CRP), erythrocyte sedimentation rate (ESR), liver enzymes, and ammonia levels were noted. Urinalysis was positive for leukocyte esterase, bacteria, and yeast. Test results for coronavirus disease 2019 and influenza A and B were negative.

**Figure 1:**
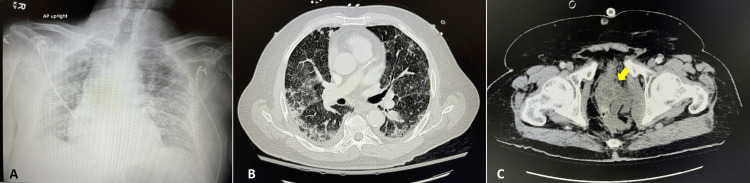
Radiography on admission. (A) Chest X-ray with bilateral diffuse interstitial and airspace opacities. (B) CT scan of the chest showing significant patchy mixed ground glass and consolidative opacities indicative of multifocal pneumonia. (C) CT scan of the abdomen with a complex multiloculated collection near the prostate extending along the lateral aspect of the rectum, which is concerning for neoplasm or abscess. CT, computed tomography

An electrocardiogram displayed a normal sinus rhythm without ST-segment elevation. A chest X-ray showed bilateral diffuse interstitial and airspace opacities (Figure 1A). A computed tomography (CT) scan of the chest revealed patchy mixed ground glass and consolidative opacities indicative of multifocal pneumonia (Figure 1B). A complex multiloculated fluid collection measuring 4.6 x 4.1 cm was identified near the prostate extending along the left lateral aspect of the rectum. A subsequent CT scan of the abdomen and pelvis confirmed this complex multiloculated fluid collection, suggestive of either a neoplasm or an abscess (Figure 1C).

**Figure 2:**
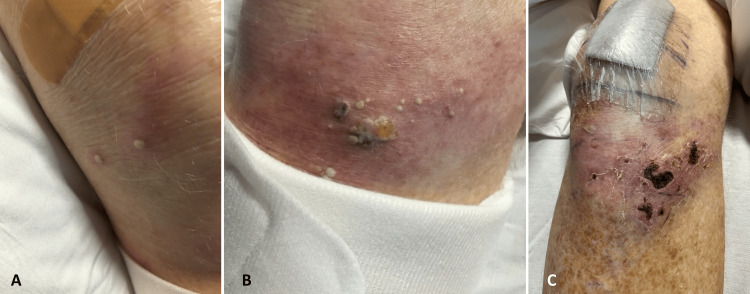
Photographs of the patient’s knee. Initial appearance of tender nodules on the right knee (A) that transformed into pustules three days post-admission (B). This image shows the early stages of infection on the patient’s knee, highlighting the localized skin changes and the formation of pustules indicative of an acute inflammatory response. The patient’s knee showed improvement (C) after initiating antibiotic therapy and surgical intervention.

**Figure 3:**
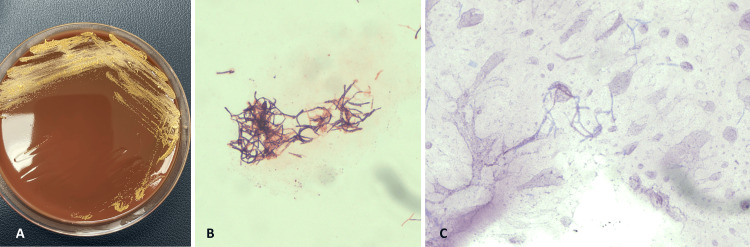
Photographs of joint fluid culture. Joint fluid culture (A) results indicating a positive identification for *Nocardia brasiliensis*. Microscopic images (B, C) showing the growth pattern typical of *Nocardia brasiliensis*, which helped in diagnosing the infection and guiding the treatment plan.

Upon admission, the patient was septic and initially treated with piperacillin-tazobactam and azithromycin. Urine cultures identified multidrug-resistant organisms (MDRO), *Klebsiella*, and *Escherichia coli*, which were susceptible to gentamicin; blood cultures showed no growth. Piperacillin-tazobactam was discontinued, and gentamicin and vancomycin were started. The urology team exchanged the suprapubic catheter. Tender nodules developed on the right knee three days post-admission, progressing to pustules (Figure 2) with moderate effusion. An X-ray of the right knee indicated soft tissue infiltration and mild osteoarthritis. Arthrocentesis yielded joint fluid positive for *Nocardia brasiliensis* (Figure 3), susceptible to amikacin, minocycline, tobramycin, and trimethoprim-sulfamethoxazole. Trimethoprim-sulfamethoxazole was initiated but discontinued upon consultation with infectious disease due to the MDRO in the urine. An orthopedic surgeon performed a right knee joint washout, and general surgery performed incision and drainage of the perirectal abscess, which yielded no significant pathogens, leading to the discontinuation of vancomycin. Antibiotic therapy was adjusted to gentamicin, cefepime, and fluconazole, leading to improvements in leukocytosis, CRP, and knee swelling. The patient was discharged to a skilled nursing facility on oral doxycycline due to elevated creatinine levels, with instructions to follow up with surgery and infectious disease specialists.

**Figure 4:**
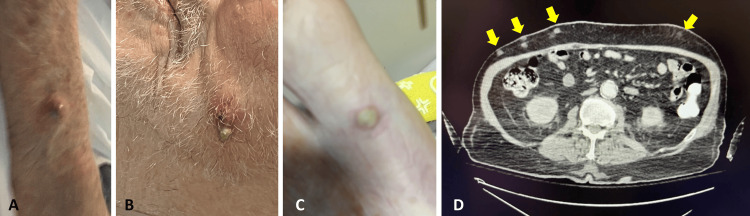
Photographs showing new appearing skin nodules on the right upper extremity (A), face (B), and left toe (C) observed during the patient’s readmission. These images illustrate the spread of infection, manifesting as purulent nodules in different body areas, signaling a potential dissemination of the pathogen or a new infection site. A follow-up CT scan (D) shows multiple subcutaneous nodules in the abdomen and pelvis. These images illustrate the internal progression of the infection, with subcutaneous nodules suggesting a deeper or disseminated infection process that extends beyond the initially observed sites. CT, computed tomography

Ten days later, the patient was readmitted with altered mentation and failure to thrive, showing a decrease in hemoglobin from 8.8 g/dL to 6.8 g/dL, requiring blood transfusion, and elevated creatinine levels at 1.7 mg/dL. A repeat brain CT scan was negative. New purulent skin nodules appeared on the right upper extremity, face, and left toe (Figures 4A, 4B, 4C). Blood, wound, and urine cultures were recollected, and empirical antibiotic therapy with meropenem was started due to renal impairment and pending culture results. Infectious disease consultation noted multiple subcutaneous nodules on a follow-up CT scan of the abdomen and pelvis (Figure 4D). Gastroenterology recommended percutaneous enterostomy tube placement for nutritional support. Subsequently, the family chose to prioritize his comfort care and decided to transition the patient to home hospice care. The right upper extremity wound culture eventually grew *Nocardia brasiliensis* and *Actinomyces*, while the urine culture showed growth of *Candida*. Blood cultures remained negative, indicating no systemic bacterial infection at that time. 

## Discussion

*Nocardia brasiliensis*, an acid-fast, gram-positive, facultative intracellular aerobic pathogen belonging to the Actinomycetes group, exhibits structural similarities to the cell wall of *Mycobacteria* species. This organism is adept at navigating dynamic soil environments, evading microbicidal actions, and developing resistance to antibiotics [6,7]. It primarily affects individuals with compromised immune systems, leading to cutaneous and localized infections stemming from trauma or skin lesions [3]. These infections may progress to other organs, including joints.

In this case, the patient’s compromised immune status, coupled with suspected direct knee inoculation following a fall, likely facilitated the *Nocardia* infection. Such localized traumatic infections can prompt an inflammatory response, resulting in abscess formation [8] or cutaneous actinomycetoma, characterized by tissue destruction and the development of fistulae and fibrotic lesions [6]. The patient’s recent TURP procedure, multifocal pneumonia, MDRO UTI, diabetes, chronic suprapubic catheter usage, and a complex multiloculated fluid collection in the deep pelvis were all contributing risk factors for joint and subsequent cutaneous involvement, heightening the risk for disseminated infection.

Diagnosing *Nocardiosis *poses significant challenges due to the absence of specific signs, symptoms, or radiological indicators [4]. While many *Nocardia* species can be identified within a few days, their inherently slow growth necessitates extended culture periods. Imaging studies, such as chest X-rays or CT scans, are critical for evaluating immunocompromised patients with symptoms like dyspnea or altered mental status where there is a suspicion of disseminated disease. Notably, this patient’s imaging did not show typical *Nocardiosis *findings, such as cavitary or non-cavitary nodules, masses, septal thickening, and centrilobular nodules [9]. The diagnosis was facilitated by gram staining and culture of joint fluid, as evidenced in Figure 2, highlighting the importance of these tests for identifying the pathogen and determining its antimicrobial sensitivity.

Trimethoprim-sulfamethoxazole (TMP-SMX) is the frontline treatment for *Nocardia* infections, either as monotherapy or in combination with other antibiotics [10]. However, its use was discontinued in this case due to the patient’s concurrent pneumonia, MDRO UTI, and perirectal abscess. The emergence of resistance to sulfonamides and TMP-SMX among *Nocardia *species complicates treatment [1]. The therapeutic arsenal typically includes TMP-SMX, amikacin, imipenem, and linezolid [4]. Management of a septic joint necessitates not only antibiotic therapy but also surgical intervention to reduce the infectious load [11]. Initially, these measures improved the condition of the patient’s right knee. However, treating a *Nocardia *infection, especially in a septic joint, requires prolonged antimicrobial therapy lasting three to six months [12]. The complexity of this case necessitated numerous adjustments in antibiotic therapy, influenced by culture sensitivities, the presence of concurrent infections, MDRO status, and renal function.

The patient’s systemic immunosuppression, exacerbated by multiple chronic conditions and recent hospitalization, likely increased his susceptibility to a *Nocardiosis* relapse. Such a relapse is characterized by the return of clinical symptoms and the re-isolation of *Nocardia *species after initial improvement [10]. Although the patient’s right knee showed signs of recovery, the appearance of skin nodules and pustules indicated cutaneous *Nocardiosis*, prompting further cultures. Nocardiosis lesions can manifest as papules, nodules, infiltrates, or abscesses [13]. The body’s defense against *Nocardia* infections primarily involves macrophages and T cells. However, chronic diseases and aging can diminish these responses [14], leading to impaired infection control and tissue healing despite antimicrobial therapy. The local immune response to *Nocardia brasiliensis* is marked by a mix of inflammatory and anti-inflammatory cytokines, fostering a milieu conducive to chronic infection and bacterial survival [6].

Despite standard treatment approaches, managing *Nocardia *infections remains challenging due to species variability, geographic prevalence differences, and specific antimicrobial susceptibilities [5]. Persistent immunosuppression further complicates treatment, often leading to a poor prognosis and significantly impacting patient and caregiver quality of life. Integrating hospice care into the patient’s treatment plan reflects the complex medical decisions required under such circumstances.

## Conclusions

*Nocardia *infection in the joint is a rare but serious condition requiring prompt recognition and treatment to prevent severe outcomes. The lack of distinct clinical signs and the slow growth of cultures complicate the diagnosis of *Nocardia* infections. In this case, the patient’s immunocompromised state, alongside trauma from a fall, was identified as the probable cause of the infection. Although a more favorable outcome might have been possible with continued or combined antibiotic regimens upon the patient’s initial discharge, managing the antibiotic therapy proved difficult due to concurrent infections, drug resistance, and renal impairment. This case underscores the importance of considering Nocardia as a potential cause of septic joints, especially in patients with similar risk profiles, and calls for further research to enhance understanding and treatment of this uncommon condition.
